# Biomarkers for Pre-Treatment Risk Stratification of Prostate Cancer Patients: A Systematic Review

**DOI:** 10.3390/cancers16071363

**Published:** 2024-03-30

**Authors:** José Pedro Sequeira, Sofia Salta, Rui Freitas, Rafael López-López, Ángel Díaz-Lagares, Rui Henrique, Carmen Jerónimo

**Affiliations:** 1 Cancer Biology & Epigenetics Group, Research Center of IPO Porto (CI-IPOP)/CI-IPOP @RISE (Health Research Network), Portuguese Oncology Institute of Porto (IPO Porto)/Porto Comprehensive Cancer Center Raquel Seruca (Porto.CCC Raquel Seruca), R. Dr. António Bernardino de Almeida, 4200-072 Porto, Portugal; jose.leite.sequeira@ipoporto.min-saude.pt (J.P.S.); sofia.salta@ipoporto.min-saude.pt (S.S.); antoniofreitas@ipoporto.min-saude.pt (R.F.); henrique@ipoporto.min-saude.pt (R.H.); 2 Epigenomics Unit, Cancer Epigenomics, Translational Medical Oncology Group (ONCOMET), Health Research Institute of Santiago de Compostela (IDIS), University Clinical Hospital of Santiago (CHUS/SERGAS), 15706 Santiago de Compostela, Spain; rafa.lopez.lopez@gmail.com (R.L.-L.); angel.diaz.lagares@sergas.es (Á.D.-L.); 3 Doctoral Program in Biomedical Sciences, ICBAS-School of Medicine & Biomedical Sciences, University of Porto (ICBAS-UP), Rua Jorge Viterbo Ferreira 228, 4050-513 Porto, Portugal; 4 Doctoral Program in Pathology and Molecular Genetics, ICBAS-School of Medicine & Biomedical Sciences, University of Porto (ICBAS-UP), Rua Jorge Viterbo Ferreira 228, 4050-513 Porto, Portugal; 5 Department of Urology & Urology Clinic, Portuguese Oncology Institute of Porto (IPO Porto)/Porto Comprehensive Cancer Center Raquel Seruca (Porto.CCC), R. Dr. António Bernardino de Almeida, 4200-072 Porto, Portugal; 6 Roche-Chus Joint Unit, Translational Medical Oncology Group (ONCOMET), Health Research Institute of Santiago (IDIS), 15706 Santiago de Compostela, Spain; 7 Centro de Investigación Biomédica en Red Cáncer (CIBERONC), ISCIII, 28029 Madrid, Spain; 8 Department of Clinical Analysis, University Hospital Complex of Santiago de Compostela (CHUS), 15706 Santiago de Compostela, Spain; 9 Department of Pathology, Portuguese Oncology Institute of Porto (IPO Porto)/Porto Comprehensive Cancer Center Raquel Seruca (Porto.CCC), R. Dr. António Bernardino de Almeida, 4200-072 Porto, Portugal; 10 Department of Pathology and Molecular Immunology, ICBAS-School of Medicine & Biomedical Sciences, University of Porto (ICBAS-UP), Rua Jorge Viterbo Ferreira 228, 4050-513 Porto, Portugal

**Keywords:** prostatic neoplasms, risk assessment, biomarkers, liquid biopsy

## Abstract

**Simple Summary:**

PCa remains a leading health concern worldwide. Serum PSA-based PCa screening led to a well-documented decreased mortality but at the cost of the increased overdiagnosis/overtreatment of indolent disease. Although various tools have been developed to predict PCa patient outcome prior to treatment, mostly based on serum PSA, the Gleason score, and clinical T stage, all have a suboptimal performance and require tissue biopsies from the prostate. To obviate that need, overcome Gleason score subjectivity and the limited specificity of serum PSA, devising more effective tools is mandatory, while also taking the opportunity to adopt minimally invasive strategies based on liquid biopsies.

**Abstract:**

Background: Prostate cancer (PCa) is one of the most frequently occurring malignancies. Although most cases are not life-threatening, approximately 20% endure an unfavorable outcome. PSA-based screening reduced mortality but at the cost of an increased overdiagnosis/overtreatment of low-risk (lrPCa) and favorable intermediate-risk (firPCa) PCa. PCa risk-groups are usually identified based on serum Prostate-Specific Antigen (PSA), the Gleason score, and clinical T stage, which have consistent although variable specificity or subjectivity. Thus, more effective and specific tools for risk assessment are needed, ideally making use of minimally invasive methods such as liquid biopsies. In this systematic review we assessed the clinical potential and analytical performance of liquid biopsy-based biomarkers for pre-treatment risk stratification of PCa patients. Methods: Studies that assessed PCa pre-treatment risk were retrieved from PubMed, Scopus, and MedLine. PCa risk biomarkers were analyzed, and the studies’ quality was assessed using the QUADAS-2 tool. Results: The final analysis comprised 24 full-text articles, in which case-control studies predominated, mostly reporting urine-based biomarkers (54.2%) and biomarker quantification by qPCR (41.7%). Categorization into risk groups was heterogeneous, predominantly making use of the Gleason score. Conclusion: This systematic review unveils the substantial clinical promise of using circulating biomarkers in assessing the risk for prostate cancer patients. However, the standardization of groups, categories, and biomarker validation are mandatory before this technique can be implemented. Circulating biomarkers might represent a viable alternative to currently available tools, obviating the need for tissue biopsies, and allowing for faster and more cost-effective testing, with superior analytical performance, specificity, and reproducibility.

## 1. Introduction

Prostate cancer (PCa) is the second most diagnosed cancer and the fifth leading cause of cancer death in men worldwide, accounting for 1,414,259 new cases and 375,304 deaths in 2020 [1]. In 2019, 2562 PCa cases were newly diagnosed and 1901 patients died from PCa in Northern Portugal [2]. Despite a relatively steady mortality over the past two decades, a trend for rising incidence has been observed, with more than 1.8 million new cases expected in 2030 [1]. Serum PSA-based screening detects ~90% of localized PCa [3]. However, PSA is not cancer-specific, leading to false-positives and consequent unnecessary biopsies, as well as an overdiagnosis of non-clinically significant PCa (ncsPCa) [4,5,6]. Additionally, PSA’s ability to monitor residual disease and predict biochemical recurrence (BCR) is rather ambiguous [7]. To improve PSA performance, tools for PCa diagnosis and the identification of clinically significant PCa, such as the prostate health index (PHI, based on −2proPSA, the percentage of free PSA, and the total PSA), 4k score (total PSA, free PSA, intact PSA, and human kallikrein 2), and PCA3 (a prostate-specific mRNA biomarker) have been reported [8]. Importantly, these three tools disclosed an area under the curve (AUC) that was superior to 0.70 [8,9]. 

PCa patients’ risk stratification is key to ensuring that adequate therapeutic decisions are made, considering the heterogenous outcomes [10]. Hence, over the years, several clinical risk-stratification tools have been proposed, among which the D’Amico Risk Classification is generally considered the gold standard. This system divides PCa patients into three different risk groups—low-risk (lrPCa), intermediate-risk (irPCa), and high-risk (hrPCa) PCa—according to serum PSA levels, Gleason score (GS) in the prostate biopsy, and clinical stage. Several upgrades to the D’Amico classification have been proposed, adding variables to the tool, such as more detailed clinicopathological information or basing classifications on risk scores or nomograms [11]. Comparing the most consensually used clinical tools [11,12,13,14,15,16,17,18,19], the Cambridge Prognostic Group’s (CPG) resulted in the highest concordance index (0.78) between the predicted and the verified PCa outcome among all of the non-nomograms tools [11,14]. According to the CPG tool, and contrary to the D’Amico system, PCa patients may be subdivided into five groups—lrPCa, favorable intermediate-risk PCa (firPCa), unfavorable intermediate-risk PCa (uirPCa), hrPCa, and very high-risk PCa (vhrPCa)—again based on GS, serum PSA, and clinical stage [14]. Nonetheless, the low specificity of serum PSA and the discordance between clinical and pathological T stage remain as weaknesses of this system. Importantly, stratification into distinct risk groups has become more important than ever, because lrPCa are usually determined to almost always be localized tumors, with a low likelihood of progression. Thus, these patients should be offered active surveillance instead of radiotherapy/prostatectomy, as no significant differences in mortality are found when comparing both strategies [20]. Moreover, identification of hrPCa’s patients allows for timely monitorization of the disease and the implementation of more accurate therapeutic decisions [21]. Nonetheless, pre-treatment risk stratification of PCa patients is complex, and several tools have been developed to overcome the inherent challenges, mainly strategies that include genomic markers. Tissue-based technologies have been reported, such as the Decipher score^®^ [22], the Prolaris^®^ cell cycle progression test [23], and the Oncotype DX^®^ Prostate Score [24]. Moreover, Grönberg and colleagues have developed a new stratification system for hrPCa—the STHLM3 [4,6,25,26]. This is based on an algorithm computing age, family history, previous biopsy results, blood biomarkers, genetic markers, digital rectal exam (DRE), and prostate volume [4,6,25,26]. Although several updates to this model have been reported, increasing its accuracy [Area Under the Curve (AUC) = 75%], its specificity remains lower than desirable (50.0%), although it has achieved a sensitivity of 84.0% [27]. Owing to its low cost-effectiveness, its use has been mostly restricted to cases with serum PSA ≥ 3 ng/mL. This, however, might lead to an increase in undetected malignancies, since only patients aged 45–75 years and with PSA > 3 ng/mL will be indicated to receive a biopsy after repeat testing validation [6]. Notwithstanding the high potential of reported tools, their high cost constitutes a barrier to its implementation in routine clinical practice [6,28]. 

Tissue biopsies remain the gold standard for PCa diagnosis [29]. Despite tissue collection from the prostate having improved due to magnetic resonance imaging-guided biopsies [30], some downsides related to tissue biopsy remain, including challenges in collecting significant amounts of material, bias due to tumor heterogeneity, and procedural issues (with adverse effects for the patient) [29,31,32]. Thus, a need to better select the patients that will benefit from a magnetic resonance imaging-guided biopsy [30,32] or from the introduction of circulating biomarkers remains. In this context, liquid biopsies emerged as alternative strategies, attracting special attention from the scientific community due to their significant potential to unveil novel biomarkers and reduce the risk of complications associated with histological biopsies [33,34,35,36]. The search for new biomarkers in liquid biopsies that could mimic the clinical/pathological variables used in D’Amico-based tools might improve the concordance between the predicted and the real PCa outcome. Moreover, unnecessary biopsies could be obviated using circulating biomarkers [36,37].

To address some of these challenges, we performed a systematic review of published data on the pre-treatment risk assessment of PCa patients using liquid biopsy strategies. This enabled us to better understand the potential of circulating biomarkers for pre-treatment risk assessment and identify gaps which must be filled before effective translation into the clinical setting.

## 2. Materials and Methods

### 2.1. Study Outcomes, Search Strategy and Selection Process

Studies that reported circulating biomarkers for pre-treatment risk stratification of PCa patients were included in this systematic review. Pubmed, MedLine, and Scopus databases were searched for publications from inception up to 24 February 2023. Grey literature or other databases were not accessed. The search strategies are provided as Supplementary Materials (Supplementary File S1). Two independent authors (JPS and SS) screened all titles and abstracts; full texts of the remaining publications were obtained, and eligibility of the publications was assessed. When discrepancies were found, a third author (RF) screened the paper. After the final selection, each study was identified with a sequential ID code to facilitate the identification by the authors.

### 2.2. Inclusion and Exclusion Criteria

Since the aim of this systematic review was to synthetize the studies which analyzed circulating biomarkers, only works that reported biomarkers evaluated in liquid biopsies were included. Studies disclosing biomarkers for risk assessment after treatment were excluded from the analysis, as well as those which did not identify the variables used for risk categorization. Reports that focused their approach on a specific subset of samples [e.g., only analyzed metastatic castration-resistant PCa (mCRPC)] were also excluded. In this systematic review, only Portuguese or English original studies were included.

### 2.3. Data Collection

The data collection was performed in a standardized form by two independent authors (JPS and SS). Variables extracted included: biomarker, type of biomarker and liquid biopsy used, risk stratification variables, method for quantification, number of samples used, and analytical variables [AUC, sensitivity, specificity, Positive Predictive Value (PPV), Negative Predictive Value (NPV), and accuracy], when available. 

### 2.4. Quality Assessment

Two authors (JPS and SS) assessed the quality of studies using the QUADAS-2 tool [38]. Bias was assessed based on: participants selection, index test description, reference test, and flow and timing as reported by Salta et al. [39]. Table 1 shows a quality assessment for all the included studies.

This systematic review was written according to the Preferred Reporting Items of Systematic Reviews and Meta-analysis of Diagnostic Test Accuracy Studies (PRISMA-DTA) guidelines [40,41] and was registered on the PROSPERO database at the Centre of Reviews and Dissemination, University of York, UK (registration number CRD42023455874) (https://www.crd.york.ac.uk/prospero/display_record.php?RecordID=455874, accessed on 1 September 2023).

**Table 1:** Studies’ quality assessment according to QUADAS-2. QUADAS items: P1 = participant selection is fully described; P2 = a consecutive or random sample of patients was selected; P3 = a case-control design was avoided; P4 = study avoided inappropriate exclusions; T1 = the index test was well described; T2 = the index test results were interpreted without knowledge of the results of the reference standard; T3 = the threshold used was pre-specified; R1 = the reference test was well described; R2 = the reference standard (groups risk) was likely to classify the target correctly; R3 = the reference standard results were interpreted without knowledge of the results of the index test; F1 = appropriate interval between index and reference test; F2 = all the patients received a reference standard (i.e., all the patients had a risk group); F3 = all the patients received the same reference standard (i.e., all the patients had a risk group according to the same stratification tool); F4 = all the patients were included in the analysis. Y = fulfilled; N = not fulfilled; U = unclear. Regarding the risk of bias, patient selection showed the highest concerns regarding applicability and risk of bias, while the other variables presented low risk of bias. The analysis of the studies’ applicability unveiled a possible concern regarding index test and reference standard.

Author, Year, Journal	Type of Biomarker	Risk of Bias																		Concerns of Applicability		
		Patient Selection					Index Test				Reference Standard				Flow and Timing					Patient Selection	Index Test	Reference Standard
		P1	P2	P3	P4	P	T1	T2	T3	T	R1	R2	R3	R	F1	F2	F3	F4	F			
Souza et al., 2020, Carcin [42]	mRNA	Y	N	N	N	High	Y	Y	N	Low	Y	N	Y	Low	U	Y	Y	N	Possible	High	Low	Possible
Connel et al., 2019, BJU Int [43]	mRNA	Y	Y	U	Y	Low	U	U	U	High	Y	Y	Y	Low	Y	N	Y	N	Possible	Low	High	Low
Van Neste et al., 2016, Eur. Urol. [44]	mRNA	Y	Y	Y	Y	Low	Y	Y	Y	Low	Y	N	Y	Low	Y	Y	N	Y	Low	Low	Low	Possible
Alvarez-Cubero et al., 2023, Int. J. Mol. Sci. [45]	mRNA	Y	N	N	U	High	Y	Y	U	Low	Y	N	Y	Low	U	N	Y	N	Possible	High	Low	Possible
Connel et al., 2021, Cancers [46]	mRNA	Y	Y	Y	Y	Low	Y	N	Y	Low	Y	N	Y	Low	Y	Y	Y	Y	Low	Low	Possible	Possible
Johnson et al., 2020, BMC Medicine [47]	mRNA	Y	Y	Y	Y	Low	Y	Y	Y	Low	Y	Y	U	Low	Y	N	U	N	High	Low	Low	Low
Rahnama’i et al., 2020, Cancer Reports [48]	mRNA	Y	Y	Y	Y	Low	Y	N	U	Possible	Y	N	Y	Low	Y	Y	Y	Y	Low	Low	Possible	Possible
Ruiz-Plazas et al., 2021, Cancers [49]	miRNA	Y	Y	Y	N	Possible	Y	N	Y	Low	Y	N	Y	Low	U	Y	Y	Y	Low	Possible	Possible	Possible
Martínez-González et al., 2021, Biomedicines [50]	miRNA	Y	U	N	U	High	Y	N	U	Possible	N	N	Y	High	U	Y	Y	U	Possible	High	Possible	High
Ramirez-Garrastacho et al., 2021, Brit. J. Can. [51]	miRNA	Y	U	U	U	High	Y	N	U	Possible	Y	N	Y	Low	U	Y	Y	Y	Low	High	Possible	Possible
Kim et al., 2021, Sci. Rep. [52]	miRNA	Y	N	N	N	High	Y	N	U	Possible	Y	Y	Y	Low	Y	Y	Y	Y	Low	High	Possible	Low
Koo et al., 2018, Small [53]	mRNA, miRNA, lncRNA	N	U	Y	U	High	Y	N	U	Possible	Y	N	Y	Low	U	Y	Y	Y	Low	High	Possible	Possible
Miyoshi et al., 2022, BMC Cancer [54]	Protein	Y	Y	Y	Y	Low	Y	Y	Y	Low	Y	N	Y	Low	U	Y	Y	Y	Low	Low	Low	Possible
Bhakdi et al., 2019, Cancers [55]	Protein	Y	Y	Y	Y	Low	Y	Y	U	Low	Y	N	Y	Low	Y	N	Y	N	Possible	Low	Low	Possible
Delkov et al., 2022, Turk J Med Sci [56]	Protein	Y	N	N	Y	Possible	Y	U	U	Possible	Y	Y	N	Low	U	Y	Y	Y	Low	Possible	Possible	Low
Mahmud et al., 2021, Anal. Chem. [57]	Protein	N	N	N	U	High	Y	N	U	Possible	Y	N	Y	Low	U	Y	Y	Y	Low	High	Possible	Possible
Ankerst at al., 2015, BMC Urology [58]	Protein	Y	N	N	Y	Possible	Y	Y	U	Low	Y	N	Y	Low	Y	N	Y	Y	Low	Possible	Low	Possible
Outzen et al., 2016, Brit. J. Nut. [59]	Protein	Y	N	N	U	High	Y	Y	Y	Low	Y	N	Y	Low	Y	Y	Y	U	Low	High	Low	Possible
Goetze et al., 2022, Clin. Prot. [60]	Protein	Y	Y	Y	Y	Low	Y	U	U	Possible	Y	N	Y	Low	Y	Y	Y	Y	Low	Low	Possible	Possible
Chiu et al., 2021, Prost. Can. Prost. Dis. [61]	Protein	N	Y	Y	U	High	Y	Y	N	Low	Y	N	Y	Low	Y	N	Y	N	Possible	High	Possible	Possible
Biggs et al., 2016, Oncotarget [62]	Protein	N	U	N	U	High	Y	U	U	Possible	Y	N	U	Possible	Y	U	U	U	High	High	Possible	Possible
Chen et al., 2022, Front. Immunol. [63]	Protein	Y	U	N	Y	Possible	Y	Y	N	Low	Y	N	Y	Low	Y	Y	Y	Y	Low	Possible	Possible	Possible
Brikun et al., 2019, Exp Hematol Oncol [64]	DNA methylation	Y	N	N	U	High	Y	U	U	Possible	Y	Y	Y	Low	U	U	U	N	High	High	Possible	Low
Connell et al., 2020, The Prostate [65]	DNA methylation	Y	Y	Y	Y	Low	Y	N	Y	Low	Y	N	Y	Low	Y	Y	Y	Y	Low	Low	Possible	Possible

## 3. Results

### 3.1. Literature Overview

**Figure 1:**
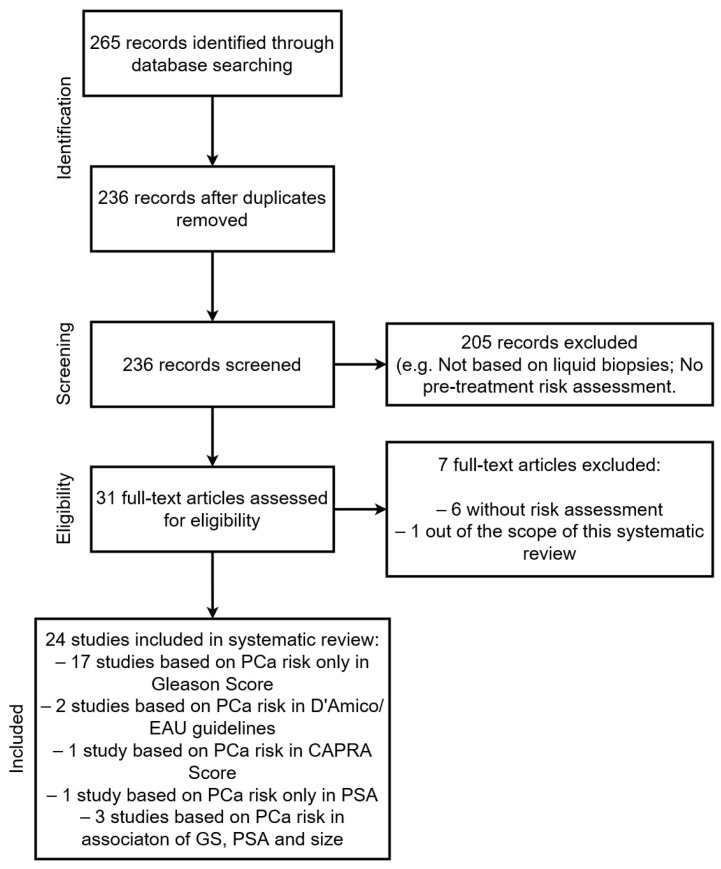
Flowchart of the literature analyzed in the study. From the 265 articles retrieved, 236 articles were screened and 31 full texts were assessed. A final list of 24 studies was included in the systematic review.

The literature search retrieved 227 papers from Scopus, 23 from PubMed, and 15 from Medline (Figure 1). 

Among these studies, thirteen based their approach on urine samples [43,44,46,47,48,51,52,53,56,57,61,64,65], five on plasma [42,45,50,59,62], four on serum [54,58,60,63], and only one used whole blood [55] and semen [49]. Although most (10/24) were based on a quantitative Polymerase Chain Reaction (qPCR) [42,44,47,48,49,50,51,52,53,64], six used mass-spectrometry technology [54,56,57,58,60,61], two analyzed the biomarkers using Nanostring [43,65], and one used Nanostring and ELISA together [46]. Digital PCR [45], chemical analysis [59], haemocytometer and fluorescence microscopy analyses [55], nanoscale flow cytometer [62], and Luminex cytokine immunoassays [63] were reported by one study each. 

The proteins DHEA [54], tCECs [55], GABA [56], Thymidine glycol [57], Sarcosine [58], Fibronectin [60], Vitronectin [60], Spermine [61], circulating prostate microparticles [62], the element Selenium [59], and the panel PHI/rPSA/fPSA/TRAIL/IL-10 [63] were reported as biomarkers for PCa risk stratification. Seven studies identified potential mRNAs that could be used as biomarkers for risk assessment [42,43,44,45,46,47,48], whereas only two studies reported DNA methylation as a tool to assess PCa pre-treatment risk [64,65]. Moreover, microRNAs (miRNAs) and the levels of miR-221-3p [49], miR-222-3p [49], miR-23c [50], miR-26a-5p [52], miR-532-5p [52], miR-99b-3p [52], miR-186-5p [51], miR-30e [51], and miR-320a-3p [51] were also assessed. Lastly, Koo and colleagues reported a panel composed of one mRNA, one miRNA, and one long non-coding RNA (lncRNA) (TMPRESS2:ERG, miR-107 and SChLAP1) [53] as potential risk biomarkers. 

According to the risk assessment tools, the majority of the studies (16 papers) categorized risk taking into account the GS/International Society of Urological Pathology (ISUP) group grading [44,45,46,48,49,50,51,52,53,54,55,57,58,61,62,63,65]. One study based their approach on GS together with T stage [42], whereas three used those two variables in a panel that also included serum PSA [47,59,60]. Furthermore, risk assessment was only categorized according to serum PSA or presence of metastasis in one study [45]. Risk assessment tools such as the CAPRA Score [64], the European Association of Urology’s (EAU) risk tool [56], and the D’Amico system [43] were used in one study each. 

### 3.2. Quality Assessment

**Figure 2:**
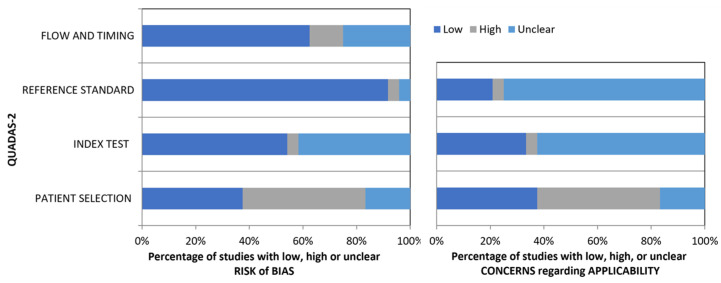
QUADAS-2 tool for assessment of the studies’ quality. Left panel represents the risk of bias and the right one the concerns regarding applicability.

The studies were evaluated for their risk of bias and concerns about their applicability using the QUADAS-2 tool (Figure 2 and Table 1). 

The risk of bias was divided into patient selection, an index test, a reference standard, and flow/timing. About 46% showed a high patient selection bias, which was mainly associated with study design, whereas most of the studies presented a low bias for the index test, reference standard, and flow/timing. Regarding concerns about applicability, about 46% showed high concerns, whereas, for the index test and reference standard, 62.5% and 75% disclosed possible concerns about applicability, respectively.

### 3.3. Biomarkers Accuracy for PCa Pre-Treatment Risk Assessment

In Table 2, the analytical performance of the circulating biomarkers is depicted. Most studies only reported the AUC, ranging from 0.60 to 0.93 [42,43,44,45,46,47,49,51,52,60,61,63,65], with the maximum value reported by Johnson and colleagues using a 25-gene panel [47]. Considering only protein biomarkers (10/24), DHEA disclosed 96.0% specificity and 98.4% PPV, but only 33.7% sensitivity [54]. TCECs were more sensitive (71.0%), but their specificity and PPV were lower (63.0% and 18.0%, respectively) [55]. The panel PHI + tPSA + fPSA + TRAIL + IL-10 depicted a higher AUC which is usually reported for protein-based biomarkers (0.92) [53], while Spermine and the Fibronectin/Vitronectin panel reported an AUC of 0.66 [60,61]. Moreover, Sarcosine and Selenium status did not disclose significant differences between risk groups, being unsuitable for use as circulating biomarkers [58,59]. Unfortunately, the remaining five studies that reported on potentially relevant biomarkers did not state AUC values [54,55,56,57,62], and six studies did not present their analytical parameters [56,57,60,61,62,63]. Concerning mRNA biomarkers, the AUC range was 0.65–0.93 [42,43,44,45,46,47]. The DLX1 and HOXC6 panel was shown to have potential for pre-treatment risk stratification by two studies [44,48]. Rahnama’i and colleagues reported a specificity and PPV of 100%, although with quite a poor sensitivity and NPV (36.8% and 8.0%, respectively) [48], whereas the opposite was reported by Van Neste et al., who found a high sensitivity and NPV (91.0% and 94.0%, respectively) but only 36.0% specificity and 27.0% PPV [44]. MRC2 and S100A4 showed potential to stratify ISUP groups, serum PSA levels, and the presence of metastasis with an AUC range of 0.60–0.67, whereas the PCA3 and S100A4 panel only showed potential to stratify the last two groups with AUCs of 0.60 and 0.68, respectively [45]. All of the analytical parameters were shown to be higher than 58% for the GOLM1 + NKX3-1 + TRPM8 panel [42]. 

Furthermore, a smaller number of miRNAs-based studies reported potential biomarkers for pre-treatment risk assessment of PCa [49,50,51,52]. The miRNAs miR-221-3p and miR-222-3p, along with the cytokine *sTWEAK,* showed 85.7% sensitivity and 76.9% specificity [49]. The remaining three miRNAs-based studies did not disclose the values for the performance parameters [50,51,52]. Only two studies were based on DNA methylation [64,65] and one study reported a panel composed of an miRNA, a mRNA and lncRNA [53]. All three of these studies reported statistical differences between risk groups, but none disclosed the results for the performance parameters.

## 4. Discussion

Managing patients across the wide spectrum of PCa, ranging from indolent to metastatic and deadly disease, requires adequate tools for clinical risk assessment. Currently available tools, such as the D’Amico and CPG methods, make use of routinely available parameters but have suboptimal performance. Thus, novel tools based on biomarkers accessible in liquid biopsies remain an unmet need [66]. These novel tools must accurately discriminate between indolent and aggressive disease, minimizing overdiagnosis and overtreatment [67,68]. 

The use of non- or minimally invasive collection methods is key for the success of this new generation of risk stratification biomarkers. In this context, liquid biopsies constitute an optimal strategy to unveil biomarkers for the detection, stratification, prognostication, and monitoring of cancer [36,45,69]. Indeed, liquid biopsies enable the analysis of several types of biomolecules, including cell-free DNA/RNA, circulating tumor cells, microvesicles, and miRNAs, among others [36,37,45,69,70,71]. Compared to tissue biopsies, this minimally invasive technology better portrays tumor heterogeneity, being faster, more cost-effective, and enabling biomarkers assessment at multiple timepoints [70,72]. Based on these advantages and considering the limitations of existing tools, we conducted a systematic review on liquid biopsy-based biomarkers reported for pre-treatment risk stratification of PCa patients. 

Focusing on the type of biomarkers assessed in the selected studies, biomarkers based on proteins, mRNA, miRNA, and DNA methylation have been reported. Among all these, proteins were clearly the most frequent type of biomarker reported, whereas DNA methylation was only addressed in two studies. In fact, all four types of biomarkers are considered to be very sensitive, enabling the detection of early events within carcinogenesis [37,39,73,74]. However, most protein-based biomarkers have not reached routine clinical practice due to failures in the validation step [75]. Regarding miRNAs, despite the well-stated potential application of miRNAs in algorithms for accurate cancer diagnosis, prognostication, and disease monitoring [76], the ubiquity of these biomolecules and the overlapping levels of them in cancer and control samples presents a major barrier to their application [77]. Moreover, notwithstanding the advantages of mRNA studies, underestimated pitfalls are reported for those biomarkers, precluding their wider use in the clinical context [78]. Concerning DNA methylation (me), this type of biomarker is the most studied epigenetic modification in humans and the first to be identified in cancer [36]. PCa-associated methylation changes are measurable in circulating cell-free DNA (in the three most studied liquid biopsies—urine, plasma, and serum), with GSTP1me being the most explored due to its high specificity (~90%) [79,80,81,82,83]. Moreover, DNA methylation-based markers may also have prognostic value [36]. Indeed, GSTP1me levels associated with ISUP grade and metastasis [84], together with RASSF2Ame, impacted non-organ-confined disease [80]. Of note, the only AUC reported in the DNA methylation-based studies included in this systematic review was 0.89, which unveils the potential of this type of marker. Despite the promising role of DNA methylation-based biomarkers, its role in PCa risk stratification remains poorly studied, entailing the need for more and larger studies. In fact, the reported results for pre-treatment risk stratification reveal that there is a long road ahead to finding a pre-treatment risk stratification biomarker that might be successfully introduced into clinical practice, and it is difficult at the moment to predict which type of marker will prove to be viable for that purpose.

There was also a diverse array of methods used to quantify the biomarkers, with 41.7% of the studies selecting qPCR, whereas only one used digital Polymerase Chain Reaction (dPCR). As previously described, dPCR allows for absolute quantification in a time- and cost-effective and accurate manner, which appears to improve biomarkers detection [37,85]. Additionally, the method’s precision and accuracy are increased by the time point of data collection [37,85,86]. This imbalance towards qPCR vs. dPCR probably derives from the longer experience which most labs have with the former technique, as dPCR is a more recent technology. Furthermore, multiplexing, which may be advantageous in this biomarker setting, is easier to accomplish in qPCR. Nonetheless, the unique characteristics of dPCR, including its superior sensitivity, make it a very attractive technology which may eventually rival qPCR. Nevertheless, more studies are required to better understand which biomarker and respective methodology will be useful for clinical practice.

Despite the great expectations for the biomarkers characterized in this systematic review, only two studies reported on performance [44,48]. Furthermore, biomarker diversity and the limited number of patients enrolled in each study without further multicentric validation remains a significative drawback to their implementation in clinical practice. Moreover, another major issue (and limitation) within the published studies results from the fact that only two studies based their approach on accepted clinical tools (the D’Amico-derived tool and a nomogram), with the majority using different strategies to those utilized in clinical practice to predict PCa patients’ outcome (e.g., only GS). The heterogeneity of clinical parameters used to stratify PCa pre-treatment risk reinforces the urgent need to unveil a highly accurate biomarker to standardize risk assessment. Nonetheless, this systematic review disclosed several interesting candidates, which are worthy of further investigation in larger and, ideally, multicentric patient cohorts.

## 5. Conclusions

Several studies have been published with the purpose of identifying novel biomarkers for pre-treatment risk stratification of PCa patients. Overall, our findings support the potential of liquid biopsies as a promising minimally invasive tool for assessing those biomarkers, obviating the limitations and disadvantages of tissue biopsies. However, current consensus on the role of circulating biomarkers for pre-treatment PCa risk-stratification considers these biomarkers as complementary rather than alternative to the existing tools, although these have clear limitations. Nonetheless, standardization of risk categories and of technical protocols is notoriously lacking and precludes the translation of research findings into clinical practice. Thus, a seemingly long road lies ahead before we can perform adequate validation studies and demonstrate the superiority of this novel strategy over the currently available tools. 

## Figures and Tables

**Table 2:** Studies’ characterization. A high heterogeneity in biomarkers and technologies used can be observed. However, it is possible to identify a tendency towards urine-based biomarkers, especially protein biomarkers, with qPCR being the most used technology. Abbreviations: AUC—area under the curve; BCR—biochemical recurrence; BPH—benign prostatic hyperplasia; csPCa—clinically significant Prostate Cancer; dPCR—digital Polymerase Chain Reaction; DRE—digital rectal exam; EAU—European Association of Urology; ISUP—International Society of Urological Pathology (ISUP) group grading; miRNA—microRNA; NA—Not applicable; ncsPCa—non clinically significant Prostate Cancer; NPV—negative predictive value; PPV—positive predictive value; pT—pathological T; qPCR—quantitative Polymerase Chain Reaction; RP—radical prostatectomy; Se—sensitivity; Sp—specificity.

Author, Year, Journal	Type of Biomarker	Biomarker	Type of Liquid Biopsies	Risk Stratification	Method	Sample	AUC	Se %	Sp %	PPV %	NPV %
Souza et al., 2020, Carcin [42]	mRNA	*GOLM1* + *NKX3-1* + *TRPM8*	Plasma	ISUP < 4 and tumor stage < pT3a vs. ISUP ≥ 4 and/or tumor stage ≥ pT3a	qPCR	60 patients who have undergone RP	0.76	85.0	58.0	61.0	83.0
Connel et al., 2019, BJU Int [43]	mRNA	PUR (Prostate Urine Risk) panel—37 genes	Urine	Normal tissue vs. D’amico low-risk vs. D’Amico intermediate-risk vs. D’Amico High Risk	Nanostring	535 first-catch post-DRE collected at diagnosis	0.72	NA	NA	NA	NA
Van Neste et al., 2016, Eur. Urol. [44]	mRNA	*HOXC6* + *DLX1*	Urine	ISUP = 1 vs. ISUP ≥ 2	qPCR	Discovery set: 519 patients; Validation set: 386 patients	DS: 0.76; VS: 0.73	DS: 91.0	DS: 36.0	DS: 27.0	DS:94.0
Alvarez-Cubero et al., 2023, Int. J. Mol. Sci. [45]	mRNA	*MRC2* + *S100A4*	Plasma	ISUP 1, 2 vs. ISUP 3, 4, 5	dPCR	20 patients with ISUP 1, 2 and 31 patients with ISUP 3, 4, 5	0.65	64.5	65.0	74.1	54.2
				PSA < 20 ng/mL vs. PSA > 20 ng/mL		26 patients with PSA < 20 ng/mL and 25 patients with PSA > 20 ng/mL	0.60	20.0	100.0	100.0	56.5
				No metastasis vs. Metastasis		12 patients without metastasis and 34 patients with metastasis	0.67	58.8	75.0	87.0	39.1
		*PCA3* + *S100A4*		PSA < 20 vs. PSA > 20		27 patients with PSA < 20 ng/mL and 29 patients with PSA > 20 ng/mL	0.60	27.6	92.6	80.0	54.3
				No metastasis vs. Metastasis		16 patients without metastasis and 38 patients with metastasis	0.68	73.7	62.5	82.4	50.0
Connel et al., 2021, Cancers [46]	mRNA	167 gene probes for cell-free RNA	Urine	ISUP = 0 vs. ISUP = 1 vs. ISUP ≥ 2	NanoString and ELISA	207 first-catch post-DRE (77 no cancer finding, 130 PCa)	0.89	NA	NA	NA	NA
Johnson et al., 2020, BMC Medicine [47]	mRNA	25-gene panel	Urine	ISUP > 2, staging ≥ T3, PSA > 20, biochemical recurrence after prostatectomy, metastasis at diagnosis/follow-up vs. other	qPCR	163 ISUP = 1, 273 ISUP = 2 and 292 ISUP ≥ 3	0.93	NA	NA	NA	NA
Rahnama’i et al., 2020, Cancer Reports [48]	mRNA	*DLX1* + *HOXC6*	Urine	ISUP < 2 vs. ISUP ≥ 2	qPCR	39 PCa (1 with ISUP < 2 and 38 with ISUP ≥ 2)	NA	36.8	100	100	4.0
Ruiz-Plazas et al., 2021, Cancers [49]	miRNA	miR-221-3p, miR-222-3p, sTWEAK	Semen	ISUP 1, 2 vs. ISUP 3, 4, 5	qPCR	97 patients who have undergone RP	0.86	85.7	76.9	NA	NA
Martínez-González et al., 2021, Biomedicines [50]	miRNA	miR-23c	Plasma	ISUP ≤ 2 vs. ISUP > 2	qPCR	60 patients with PSA ≥ 4 ng/mL meeting the criteria for undergoing a prostate biopsy	NA	NA	NA	NA	NA
Ramirez-Garrastacho et al., 2021, Brit. J. Can. [51]	miRNA	Hsa-miR-186-5p + PSA	Urine	ISUP 1 vs. 2 vs. 3	qPCR	60 PCa (20 from each ISUP group)	0.78	NA	NA	NA	NA
		Hsa-miR-30e + PSA					0.74	NA	NA	NA	NA
		Hsa-miR-320a-3p + PSA					0.77	NA	NA	NA	NA
Kim et al., 2021, Sci. Rep. [52]	miRNA	ExomiR-26a-5p	Urine	ISUP = 2 BCR vs. ISUP = 2 non-BCR	qPCR	Discovery set: 21 non-BCR, 6 BCR; Validation set: 28 non-BCR, 26 BCR	0.67	NA	NA	NA	NA
		ExomiR-532-5p					0.67				
		ExomiR-99b-3p					0.67				
Koo et al., 2018, Small [53]	mRNA, miRNA, lncRNA	*TMPRSS2:ERG* + miR-107 + SChLAP1	Urine	ISUP ≤ 2 vs. ISUP > 2	qPCR	18 PCa samples (10 with ISUP ≤ 2 and 8 with ISUP > 2) and 2 HD	NA	NA	NA	NA	NA
Miyoshi et al., 2022, BMC Cancer [54]	Protein	*DHEA*	Serum	BPH + ISUP ≤ 2 vs. ISUP > 3	LC-MS/MS	203 patients with PSA levels < 10 ng/mL	NA	33.7	96.0	98.4	16.9
Bhakdi et al., 2019, Cancers [55]	Protein	*tCECs*	Whole Blood	1st definition: csPCa vs. ncsPCa (ISUP ≥ 2 vs. ISUP < 2). 2nd definition: csPCa vs. ncsPCa (ISUP ≥ 3 vs. ISUP <3 )	Haemocytometer and fluorescence microscopy	146 PCa patients	NA	1st: 75.0 2nd: 71.0	1st:67.0 2nd:63.0	1st:32.0 2nd:18.0	1st:93.0 2nd:95.0
Delkov et al., 2022, Turk J Med Sci [56]	Protein	*GABA*	Urine	EAU stratification system: High-risk vs. Intermediate risk. High-Risk vs. Low-Risk	HPLC-MS/MS	101 PCa patients and 52 controls	NA	NA	NA	NA	NA
Mahmud et al., 2021, Anal. Chem. [57]	Protein	*Thymidine glycol*	Urine	ISUP = 1 vs. ISUP ≥ 2	PSI-MS	40 PCa patients (10 from each ISUP) and 10 HD	NA	NA	NA	NA	NA
Ankerst at al., 2015, BMC Urology [58]	Protein	*Sarcosine*	Serum	ISUP = 1 vs. ISUP ≥ 2	HPLC-electrospray ionization mass spectrometry	246 cancer cases and 251 age-matched non-cancer cases	not significant. *p*-value > 0.05	NA	NA	NA	NA
Outzen et al., 2016, Brit. J. Nut. [59]	Protein	*Selenium*	Plasma	>T3 or ISUP > 2 or N1 or M1, or “regional/distant” extent of disease or PSA > 15 VS High-grade PCa—ISUP > 4	chemical analyses	784 cases (525 advanced PCa, 170 high-grade PCa, 89 low-grade PCa)	not significant. *p*-value > 0.05	NA	NA	NA	NA
Goetze et al., 2022, Clin. Prot. [60]	Protein	*Fibronectin* + *vitronectin*	Serum	total PSA = 4–10, tumor stage = pT2, and ISUP ≤ 1 vs. total PSA > 10, tumor stage = pT3, and ISUP ≥ 2	Development phase: MS-GUIDE/Validation: ELISA	Discovery set: 78 patients; validation set: 263 patients	0.66	NA	NA	NA	NA
Chiu et al., 2021, Prost. Can. Prost. Dis. [61]	Protein	*Spermine*	Urine	ISUP 1 vs. ISUP 2, 3, 4, 5	UPLC-MS/MS	600 Patients	0.82 (Spermine + prostate volume + PSA + age + DRE). 0.66 (spermine)	NA	NA	NA	NA
Biggs et al., 2016, Oncotarget [62]	Protein	*Circulating Prostate Microparticles*	Plasma	ISUP ≤ 2 vs. ISUP > 2	Nanoscale flow cytometry	Healthy volunteers = 22, BPH = 156, localized PCa = 256, CRPC = 67	NA	NA	NA	NA	NA
Chen et al., 2022, Front. Immunol. [63]	Protein	*PHI* + tPSA + fPSA + *TRAIL* + IL-10	serum	ISUP = 1 vs. ISUP ≥ 2	Luminex cytokine immunoassays	79 aggressive PCa and 209 indolent PCa or BPH	0.92	NA	NA	NA	NA
Brikun et al., 2019, Exp Hematol Oncol [64]	DNA methylation	32 markers	Urine	CAPRA risk	qPCR	15 group 1 (low), 18 group 2 (high)—DRE samples	NA	NA	NA	NA	NA
						10 group 1 (low), 18 group 2 (high)—first void samples	NA	NA	NA	NA	NA
Connell et al., 2020, The Prostate [65]	DNA methylation	*GSTP1*, *SFRP2*, *IGFBP3*, *IGFBP7*, *APC*, *PTSG2* and 167 gene-probes of cell free RNA	Urine	ISUP = 1 vs. ISUP = 2 vs. ISUP ≥ 3	NanoString	297 first-catch post-DRE (77 no cancer finding, 120 PCa)	0.89	NA	NA	NA	NA

## Data Availability

The original contributions presented in the study are included in the article/supplementary material, further inquiries can be directed to the corresponding author.

## Supplementary Materials

The following supporting information can be downloaded at: https://www.mdpi.com/article/10.3390/cancers16071363/s1, Supplementary Materials S1: Search strategies of the systematic review and abbreviations.
