# Supplementary material for: Biomarkers for Pre-Treatment Risk Stratification of Prostate Cancer Patients: A Systematic Review
Source: Cancers (Basel). 2024 Mar 30;16(7):1363. doi: 10.3390/cancers16071363 (PMC11011064; doi:10.3390/cancers16071363)
Supplement: Supplementary file 1 [file cancers-16-01363-s001.zip › cancers-2916742-supplementary.pdf]

Search strategies of the systematic review

PubMed search strategy (23 papers retrieved)

For Pubmed search, the following strategy was used: ((prostatic neoplasms[MeSH Terms]) OR (Prostate Cancer[Title/Abstract]) OR (Prostate Tumor[Title/Abstract])) AND ((Biomarkers, Tumor[MeSH Terms]) OR (biomarkers[MeSH Terms]) OR (biomarker[Title/Abstract])) AND ((Liquid Biopsy[MeSH Terms]) OR (body fluids[MeSH Terms])) AND ((risk assessment[MeSH Terms]) OR (risk[MeSH Terms]) OR (pre-treatment risk[Title/Abstract]) OR (stratification[Title/Abstract]) OR (risk stratification[Title/Abstract]))

Scopus search strategy (227 papers retrieved)

For Scopus search, the following strategy was used, restricted to Title, abstract, and keywords: ((prostatic neoplasms) OR (Prostate Cancer) OR (Prostate Tumor)) AND ((Tumor Biomarker) OR (biomarkers) OR (biomarker)) AND ((Liquid Biopsy) OR (body fluids)) AND ((risk assessment) OR (risk) OR (pre-treatment risk) OR (stratification) OR (risk stratification))

MEDLINE/ESBCO search strategy (15 papers retrieved)

For Medline/ESBCO search, the following strategy was used: ((MH prostatic neoplasms) OR (AB Prostate Cancer) OR (AB Prostate Tumor)) AND ((MH Biomarkers, Tumor) OR (MH biomarkers) OR (AB biomarker)) AND ((MH Liquid Biopsy OR (MH body fluids)) AND ((MH risk assessment) OR (MH risk) OR (AB pre-treatment risk) OR (AB stratification) OR (AB risk stratification))

Abbreviations

AUC – Area Under the Curve

BCR – Biochemical Recurrence

BPH – Benign Prostatic Hyperplasia

CPG – Cambridge Prognostic Groups

csPCa – clinically significant Prostate Cancer

dPCR – digital Polymerase Chain Reaction

DRE –Digital Rectal Exam

EAU – European Association of Urology

firPCa – favorable intermediate-risk Prostate Cancer

GS – Gleason Score

hrPCa – high-risk Prostate Cancer

irPCa – intermediate-risk Prostate Cancer

ISUP – International Society of Urological Pathology

lncRNA – long non-coding RNA

lrPCa – low-risk Prostate Cancer

mCRPC – metastatic castration-resistant Prostate Cancer

miRNA – microRNA

ncsPCa – non-clinically significant PCa

NPV – Negative Predictive Value

PCa – Prostate cancer

PPV – Positive Predictive Value

PRISMA-DTA – Preferred Reporting Items of Systematic Reviews and Meta-analysis of Diagnostic Test Accuracy Studies

PSA – Prostate-Specific Antigen

pT – pathological T

qPCR – quantitative Polymerase Chain Reaction

RP – Radical Prostatectomy

Se – Sensitivity

Sp – Specificity

uirPCa – unfavorable intermediate-risk Prostate Cancer

vhrPCa – very high-risk Prostate Cancer
